# Rare Coagulation Disorders: A Retrospective Analysis of 156 Patients in Turkey

**DOI:** 10.5505/tjh.2012.02418

**Published:** 2012-03-05

**Authors:** Tunç Fışgın, Can Balkan, Tiraje Celkan, Yurdanur Kılınç, Meral Türker, Çetin Timur, Türkiz Gürsel, Emin Kürekçi, Feride Duru, Alphan Küpesiz, Lale Olcay, Şebnem Yılmaz, Ünsal Özgen, Ayşegül Ünüvar, Hale Ören, Kaan Kavaklı

**Affiliations:** 1 Ondokuz Mayıs University, School of Medicine, Department of Pediatric Hematology, Samsun, Turkey; 2 Ege University, School of Medicine, Department of Pediatric Hematology, İzmir, Turkey; 3 İstanbul University, Cerrahpaşa School of Medicine, Department of Pediatric Hematology and Oncology, İstanbul, Turkey; 4 Çukurova University, School of Medicine, Department of Pediatric Hematology, Adana, Turkey; 5 Tepecik Research and Education Hospital, Department of Pediatric Hematology, İzmir, Turkey; 6 Göztepe Training and Research Hospital, Department of Pediatric Hematology, İstanbul, Turkey; 7 Gazi University, School of Medicine, Department of Pediatric Hematology, Ankara, Turkey; 8 Gülhane Military Medical Academy, Department of Pediatric Hematology, Ankara, Turkey; 9 Akdeniz University, School of Medicine, Department of Pediatric Hematology, Antalya, Turkey; 10 Dr. Abdurahman Yurtarslan Oncology Training and Research Hospital, Department of Pediatric Hematology, Ankara, Turkey; 11 Dokuz Eylül University, School of Medicine, Department of Pediatric Hematology, İzmir, Turkey; 12 İnönü University, School of Medicine, Department of Pediatric Hematology, Malatya, Turkey; 13 Istanbul University, İstanbul School of Medicine, Department of Pediatric Hematology and Oncology, İstanbul, Turkey

**Keywords:** Rare coagulation deficiencies, clinical findings, Laboratory data

## Abstract

**Objective:** To retrospectively evaluate the clinical findings, laboratory data, management, and outcome in a group ofTurkish children diagnosed with rare coagulation deficiencies (RCDs) between January 1999 and June 2009.

**Material and Methods:** The Turkish Society of Pediatric Hematology-Hemophilia-Thrombosis-Hemostasissubcommittee designed a Microsoft Excel-based questionnaire for standardized data collection and sent it to participatinginstitutions.

**Results:** In total, 156 patients from 12 pediatric referral centers were included in the study. The cost common RCDswere as follows: FVII (n = 53 [34%]), FV (n = 24 [15.4%]), and FX (n = 23 [14.7%]) deficiency. The most common initialfinding in the patients was epistaxis, followed by ecchymosis, and gingival bleeding.

**Conclusion:** Initial symptoms were mucosal bleeding, and fresh frozen plasma (FFP) and tranexamic acid werethe most commonly used treatments. We think that prophylactic treatment used for hemophilia patients should beconsidered as an initial therapeutic option for patients with rare factor deficiencies and a severe clinical course, and forthose with a factor deficiency that can lead to severe bleeding.

## Abstract (tr)

**Amaç:** Türkiye’de son 10 yılda nadir koagülasyon eksikliği olan çocukların klinik bulguları, laboratuvar değerleri ve tedavilerini geriye yönelik olarak değerlendirdik.

**Gereç ve Yöntemler:** Türk Pediatrik Hematoloji Derneği, Tromboz-Hemostaz-Hemofili Alt Komitesi tarafından standardize edilmiş excel sorgulama dosyaları her merkeze gönderilerek yanıt veren merkezlerin verileri toplandı.

**Bulgular:** Oniki çocuk hematoloji merkezinden toplam 156 hasta bu çalışmaya alındı. Çalışmamızda en sık görülen nadir koagülasyon bozuklukları FVII (n: 53 olgu, %34), FV (n: 24 olgu, %15.4), FX (n: 23 olgu, %14.7) olarak dağılım gösterdi. Tüm nadir koagülasyon bozuklukları içinde en sık görülen başlangıç semptomları sırası ile burun kanaması, ekimoz ve diş eti kanamasıydı.

**Sonuç:** Mukozal kanamalar ilk semptomları oluştururken, halen en sık kullanılan tedavi seçenekleri ise taze donmuş plazma ve treneksamik asitti. Ağır klinik semptomları olan ve şiddetli kanamaya neden olabilecek nadir koagülasyon bozukluklarının erken dönemlerinde de hemofili hastalarında kullandığımız proflaktik tedavinin düşünülmesi gerektiğini öneriyoruz.

## INTRODUCTION

Rare coagulation deficiencies (RCDs) of childhood arecommonly inherited in an autosomal recessive pattern,and include factor I (FI), FII, FV, FVII, FX, and FXIII deficiency[1,2]. RCDs, as the term implies, are rarely encountered.A prevalence as high as 1/20,000 was reported inpopulations with consanguineous marriage; however, theestimated prevalence of RCDs is 1/500,000-1/2,000,000[1,2]. Although most RCD patients are asymptomatic,patients present with bruising, and mucosal and dermalbleeding, such as ecchymosis, epistaxis, gingival bleeding,and menorrhagia [1,2,3,4,5]. In addition, hemarthrosis,hematoma, and central nervous system bleeding may beseen [2,3]. The frequency and severity of bleeding in RCDpatients vary, and are related to the type and level of factordeficiency. While intracerebral hemorrhagia may bemostly seen in FX and FXIII, afibrinogenemia, and FVIIdeficiency, musculoskeletal bleeding (hemarthrosis andhematoma) more frequently occurs in afibrinogenemia,and FX, FXI, and FXIII deficiency [2,6,7,8].

There are 2 treatment options for RCDs—on demandand prophylactic. On demand treatment is usually administeredto RCD patients by hematologists because of therare symptoms of bleeding, and generally includes freshfrozen plasma (FFP), epsilon-aminocaproic acid (EACA),tranexamic acid, cryoprecipitate, activated or non-activatedprothrombin complex concentrates (aPCC andPCC, respectively), and recombinant factor VIIa [1,2,3,4,5,6,7,8,9,10]. Treatment strategies for RCDs are largely based on theseverity and localization of bleeding. Interestingly, EACAor tranexamic acid treatment alone controlled the bleedingin almost 33% of patients with RCDs [1,2]. As such, the present study aimed to retrospectively evaluate the clinicalfindings, laboratory data, management, and outcomein 156 Turkish children diagnosed with RCDs during a10-year period.

## MATERIALS AND METHODS

We retrospectively analyzed initial clinical and laboratoryfindings, management, and outcome data for 156 childrenwith RCDs obtained from 12 pediatric referral centersin Turkey. The Turkish Society of Pediatric HematologyHemophilia - Thrombosis - Hemostasis subcommitteedesigned a Microsoft Excel-based questionnaire for standardizeddata collection and sent it to the participatinginstitutions. Ethics Committee approved the study. All children were diagnosed with an RCD betweenJanuary 1999 and June 2009. RCDs were diagnosed basedon bleeding history, and prolonged prothrombin time(PT) and/or activated partial thromboplastin time (APTT)and low coagulation factor level (FVII, FV, FX, FXIII, andFXI <40%, and FI <100 mg dL^-1^) [1,2,3,4]. Unfortunately,genetic diagnosis could have been performed in very limited patients. Patient age, medical history, clinical findings, laboratorydata (factors levels), treatment, and prognosis wererecorded. Demographic, laboratory, and clinical data wereanalyzed using descriptive statistics. Patients with the 3most common RCDs were grouped, as follows, to comparetheir clinical and laboratory findings: group 1 (FVIIdeficiency), group 2 (FV deficiency), and group 3 (FXdeficiency). Group results were compared using student’st test and the Mann-Whitney U test. The level of statisticalsignificance was set at P < 0.05. Statistical analysis wasperformed using SPSS v.13.0.

## RESULTS

The distribution of RCDs was as follows: FVII deficiency(n = 53 [34%]), FV deficiency (n = 24 [15.4%]),FX deficiency (n = 23 [14.7%]), FXIII deficiency (n = 16[10.3%]), FI deficiency (n = 15 [9.6%]), FXI deficiency (n= 13 [8.3%]), FXII deficiency (n = 6 [3.8%]), plasminogendeficiency (n = 3 [1.9%]), and combined FV-VII deficiency(n = 3 [1.9%]). Among all the patients, the most commoninitial finding was epistaxis, followed by ecchymosis, an dgingival bleeding.FVII deficiency was the most common RCD (n = 53[34%]). Median age at the time of diagnosis in the 53patients (19 girls and 34 boys) with FVII deficiency was72 months (range: 0.1-180 months). The most frequentlyobserved bleeding symptoms were epistaxis, gingivalbleeding, and ecchymosis, and the parental consanguinityand positive family history rates were 24.5% and 20.8%,respectively. Molecular diagnosis was performed in only 2of the 156 patients. FFP and recombinant FVIIa (rFVIIa)were the most common treatment choices in the patientsthat received on demand treatment; only 1 patient receivedprophylactic treatment with recombinant FVIIa. The mortalityrate was 3.8% (n = 2) and the cause of mortality wasintracranial bleeding in both cases.Median age at the time of diagnosis was significantlyhigher in group 1 (72 months) than in group 2 (30 months) and group 3 (9 months). In all, 11 of the 53patients in group 1 were treated with FFP. RecombinantFVIIa was administered to only 8 of 53 patients Despitethe development of intracranial bleeding in 7 patients and 2 patients died prophylaxis was started to only 1 patientafter intracranial bleeding. Patient characteristics accordingto the type of RCD (FVII, FV, FX, FI, FXIII, and FXIdeficiency) is shown in Table 1. Comparison of the 3 mostcommon RCDs (FVII, FV, and FX deficiency; groups 1, 2 and 3, respectively) is shown in Table 2 and the distributionof intracranial bleeding according to the type of RCDis shown in Table 3.

## DISCUSSION

Clinical manifestations of RCDs vary from mild tosevere, depending on the type of and level of factor deficiency,and underlying molecular defects [1,2]. Bleedingin patients with RCDs is generally a rare occurrence.Moreover, primarily hematologists follow-up and treat RCD patients, the management of whom is difficult due tolimited experience and lack of clear treatment guidelines.Although historically only tranexamic acid and FFP weretreatment options available to hematologists, currently,plasma-derived factor concentrates and recombinant factorconcentrates are also available [1,2,6,7,8,9,10,11,12].

In countries in which consanguineous marriage is common,such as Iran, Turkey, and India, the frequency ofrecessively inherited coagulation deficiencies is high and the frequency of inherited deficiencies of fibrinogen, prothrombin,FV, FVII, FV+FVIII, FX, and FXIII is 3-7-foldhigher in Iran than in Italy and the UK, as reported byPeyvandi et al. [13], who also reported that FXI deficiencywas more frequent in the UK than expected, probablybecause of its population of Ashkenazi Jews. In thepresent study FXI deficiency was the least common RCD,as in Iran [13]. Consistent with the literature, the mostcommon initial finding in the present study’s patients wasepistaxis, followed by ecchymosis and gingival bleeding[1,2,3,4]; however, the high rate of intracranial bleeding inthe patients with FI and FXIII deficiency (26% and 25%,respectively) and menorrhagia as the most frequent typeof bleeding (38%) in the patients with FXI deficiency arequite remarkable findings.

The diagnosis of FVII deficiency is often made duringchildhood, but symptoms of bleeding begin during theneonatal period in those with severe deficiency [9,11,14].Mucosal bleeding detected most frequently in our serieswere in accordance with the pattern reported for FVII deficiencyas epistaxis and gingival bleedings. Median age atthe time of diagnosis was significantly higher in group 1than in group 3, probably due to factor levels. 20.8% ofpatients in the present study were treated with FFP; rFVIIawas used in a limited number of patients (15,1%). Despite the development of intracranial bleeding and death inseven patients, prophylaxis was initiated in only 1 patient.rFVIIa is recommended for the treatment of FVII deficiency,as the half-life of this factor is very short. Frequentuse of FFP may create fluid overload; in an effort to avoidthis, FFP may be administered less frequently than neededto provide adequate bleeding control. Although rFVIIa hasbeen available in Turkey since 2003 the present resultsshow that most of the hematologists at the institutions thatparticipated in the study did not use this treatment option,which could have been due to its high cost. This result andvery limited implementation of prophylaxis are the issuesthat should be thought on it.

Most patients with FV deficiency present with epistaxisand mucosal bleeding, usually diagnosed before the age of6 years; however, patients as old as 62 years with intracranialbleeding have been reported [10,15,16]. In the presentstudy’s patients with FV deficiency mean age at the timeof diagnosis was 3.5 years, and gingival and nose bleedingwere the most common types of bleeding. As no recombinantor FV concentrate products are commercially unavailable,FFP or antifibrinolytic treatment was used in 50% ofthe present study’s patients, as previously reported [2].

Menagatti et al. reported the most frequent symptomof bleeding in patients with FX deficiency is epistaxis [17],and Peyvandi et al. reported that central nervous systembleeding, hematoma, and hemarthrosis are common [2]. It is known that more serious symptoms of bleeding occur inpatients with FX deficiency than in those with other rarefactor deficiencies [2,5]. In the present study the most frequentlyencountered symptom of bleeding was epistaxis,followed by ecchymosis, and hemarthrosis. Mean age ofthe patients at the time of diagnosis was significantly lowerin those with FVII deficiency than in the other patients.In accordance with the literature, intramuscular bleeding and hemarthrosis occurred more frequently in the patientswith FX deficiency, as compared to those with other factordeficiencies, and significantly more frequent hematomaand hemarthrosis rates compared to FVII deficient group.We observed a significantly higher rate of subconjunctivalbleeding, which is a rarely observed form of bleeding, in thepatients with FX deficiency than in those with FVII deficiency.In addition, the rate of prophylactic treatment wassignificantly higher in the patients with FX deficiency thanin those with FVII deficiency. High rate of prophylaxis inFX deficiency, diagnosed at young ages with serious bleedingsymptoms, is a satisfactory and acceptable approach.

Anemia occurs in 19%-49% and erythrocyte transfusionis required by 10%-20% of patients with rare factor deficiencies [1]. We did not make an inquiry for theseparameters in our series. Despite the reports on post-circumcisionbleeding in some series as the most frequentdiagnostic finding in rare factor deficiencies it was notwithin the first three bleeding symptoms of our threegroups (FVII, FV, FX deficiencies) [1]. Acharya et al.reported a central nervous system bleeding-related morbidityrate of 9%-22% in patients with RCDs [1].

In the present study 15% (24/156) of the patients hadintracranial bleeding. Whereas intracranial bleeding was notobserved in any of the patients with FXII deficiency, 26%(the highest rate) of those with FI deficiency had intracranialbleeding; despite this fact, FI concentrate was administeredto only 3 patients while FFP (78%) and tranexamicacid were used more frequently as treatment options. Themortality rate due to intracranial bleeding in the presentstudy was 3% (5/156)—2 of the patients that died hadFVII deficiency, 2 had fibrinogen deficiency, and 1 had FXdeficiency. Ten of the 19 patients that survived intracranialbleeding were using prophylaxis. Despite the intracranialbleeding, lack of prophylactic treatment including FFP innearly half of the patients (9/19) makes us think that it isnecessary to review our treatment options in these patients.

While bleeding is expected in patients with rare factordeficiencies, the development of thrombus is rarelyobserved [18]. In the present study 1 patient with FXIIIdeficiency was hospitalized with the presumptive diagnosisof intracranial bleeding, but was eventually diagnosedas sinovenous thrombosis. The patient was heterozygousfor factor V Leiden mutation and was treated successfullywith low molecular weight heparin [18]. Patients withboth FV and FVIII deficiency are generally diagnosed withmild or moderate mucosal bleeding [2,19]. Additionally,patients diagnosed with post circumcision bleeding ormenorrhagia have been reported [21,22]. Mucosal bleedingswere predominant in our 3 cases with FV-FVIII combineddeficiency.

A frequent finding in patients with plasminogen deficiencyis conjunctivitis, which can involve the cornea[22,23]. A pseudomembrane may develop on the gums,ears, and the respiratory tract, in addition to the eyes,and hydrocephalus may accompany the clinical picture[22,23]. In accordance, the presenting complaint in 2 ofthe present study’s patients was ligneous conjunctivitis,and their ophthalmologic findings recurred and persisteddespite intermittent surgical treatment.

In conclusion, in the majority of patients with rare factor deficiencies the initial symptom was mucosal bleeding,and FFP and tranexamic acid were the most commonly used therapeutic options. Difficulty diagnosing these rare factor deficiencies, especially at the molecular level, is amajor problem in Turkey. Genetic diagnosis was made in only 2 of the present study’s patients (both treated at the same center) via correspondence with a center abroad.Thus, diagnosis of all the other patients was based on medical history, symptoms, and coagulation testing; therefore,nationally defining and determining the centers that could perform diagnosis of genetic studies on this issue and providing these centers with financial support seem to be the most probable resolution of this problem. Lack of specific treatment guidelines that are agreed upon by the centers is another problem. We observed that episodic treatment was initiated according to the presence of symptoms,whereas prophylaxis was used on a very limited basis event hough specific recombinant products for treating the deficiencies were commercially available. We think that the prophylactic treatment we use for hemophilia patients should be considered as an initial therapeutic option for patients with rare factor deficiencies and a severe clinical course, and in those with factor deficiencies that can leadto severe bleeding.

## CONFLICT OF INTEREST STATEMENT

The authors of this paper have no conflicts of interest, including specific financial interests, relationships, and/ or affiliations relevant to the subject matter or materials included.

## Figures and Tables

**Table 1:**
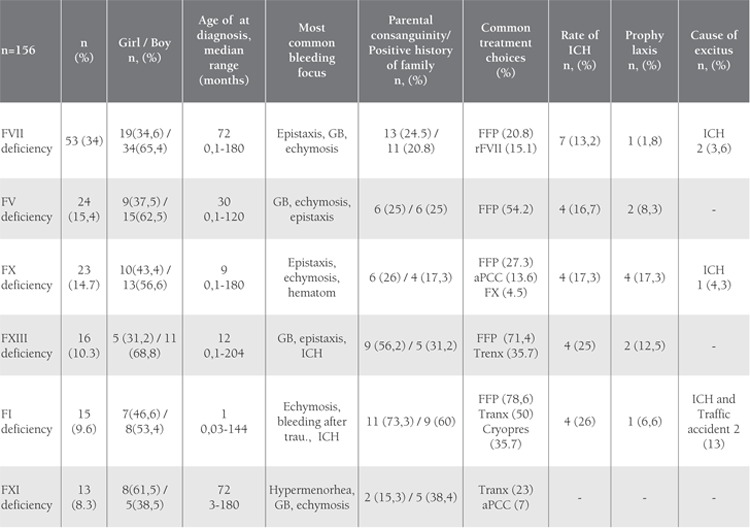
Detailed data of rare Coagulation Disorders

**Table 2:**
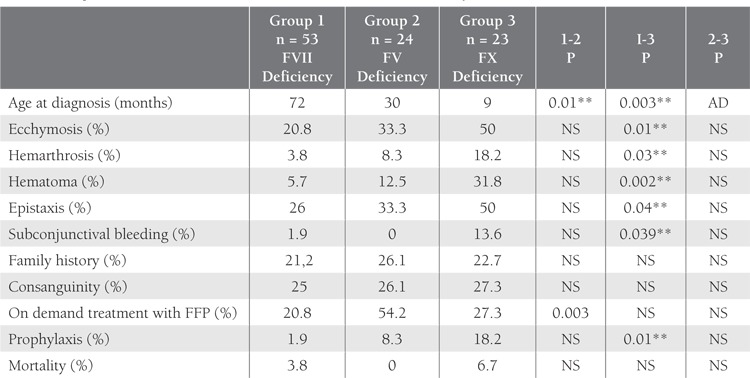
Comparison of the 3 Most Common RCDs (FVII, FV, and FX Deficiency)

**Table 3:**
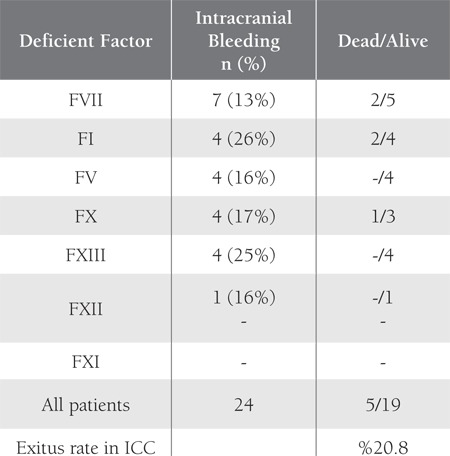
Distribution of Intracranial Bleeding
